# Supplementary material for: Estimating the volume and age of water stored in global lakes using a geo-statistical approach
Source: Nat Commun. 2016 Dec 15;7:13603. doi: 10.1038/ncomms13603 (PMC5171767; doi:10.1038/ncomms13603)
Supplement: Supplementary Information — Supplementary Figures 1-6, Supplementary Tables 1-5, Supplementary Discussion and Supplementary References. [file ncomms13603-s1.pdf]

SUPPLEMENTARY FIGURES

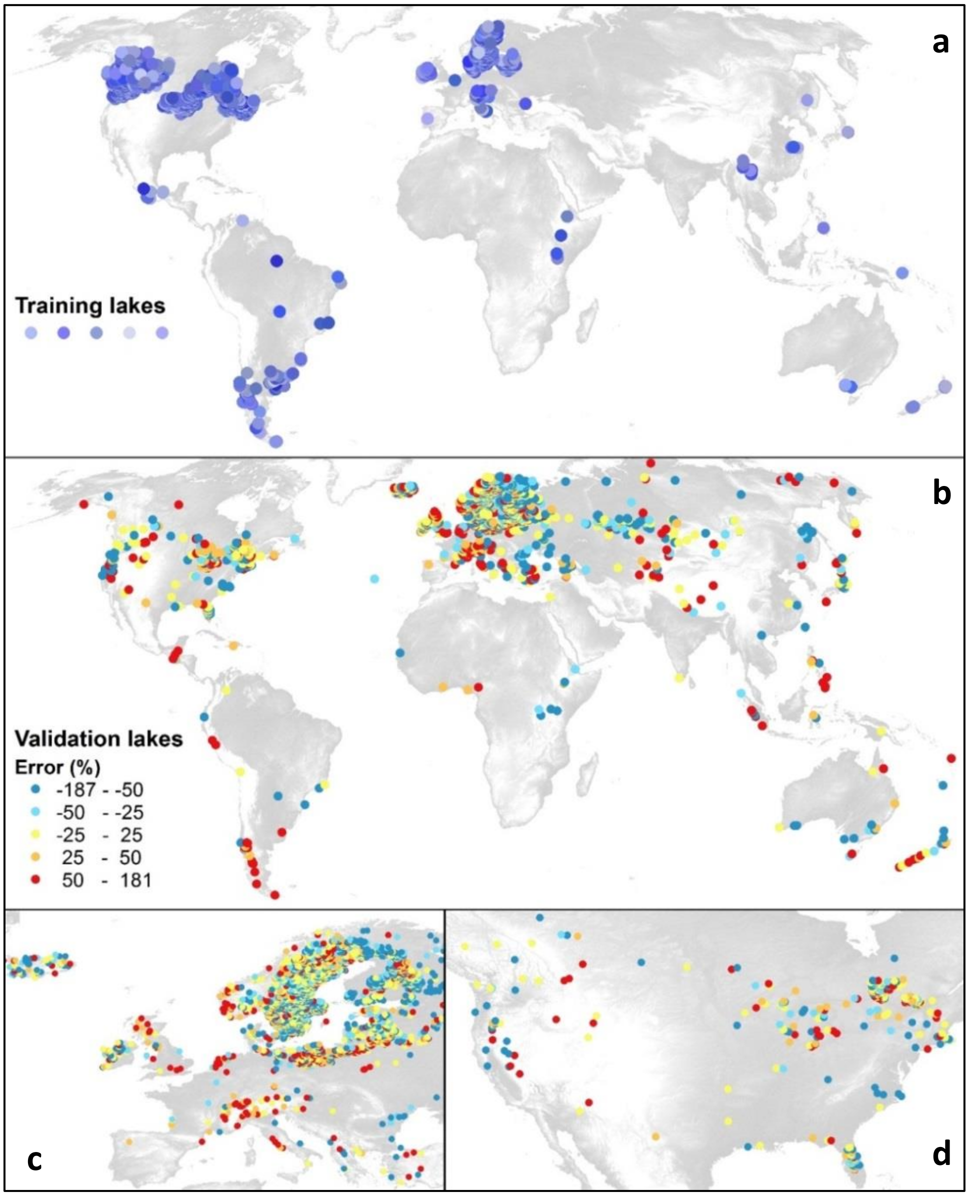

Supplementary Figure 1. Training and validation lake datasets.

Each point represents a lake (total 12,150 records). Global panel (a) shows distribution of training data (7049 records). Global panel (b) and regional panels (c-d) show error estimates in the prediction of lake volumes calculated by comparing Model 5 results with independent validation data (5101 records). The error estimate is calculated in analogy to the Symmetric Mean Absolute Percent Error (SMAPE) as $(V_{reference} – V_{predicted}) / ((V_{reference} + V_{predicted}) / 2)$. Negative values (light and dark blue) indicate lakes for which the empirical measure of the lake volume is smaller than the volume predicted by the model (overestimation). Positive values (orange and red) indicate lakes for which the empirical measure of the lake volume is larger than the volume predicted by the model (underestimation).

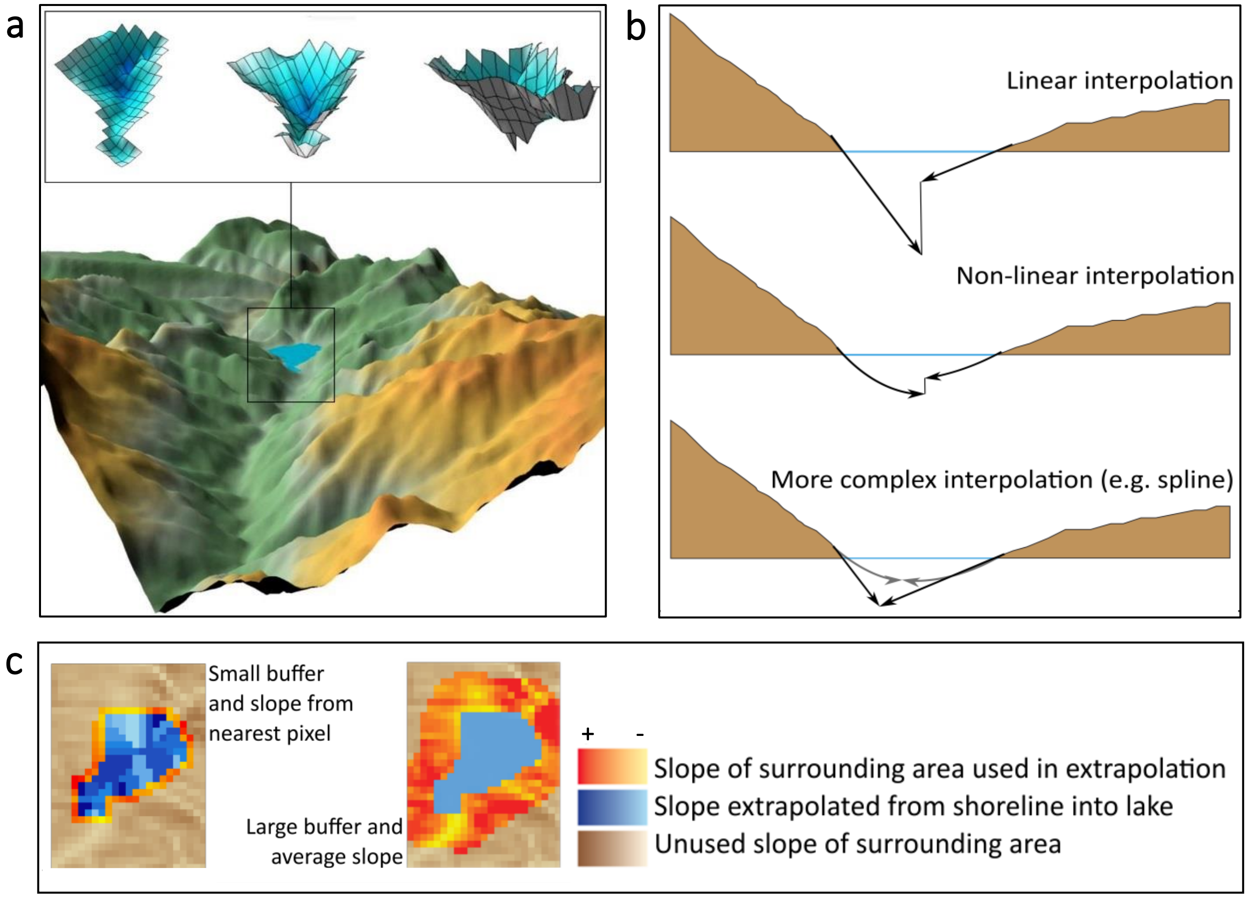

Supplementary Figure 2. Different approaches for modeling the 'topographic depth' of a lake by analyzing the surrounding terrain information.

Panel (a) shows a 3-D representation of the output of bathymetry modelling through slope expansion. Panel (b) shows different cross-sections of alternative methods to extrapolate lake bathymetry from surrounding topography using slopes. Panel (c) shows two examples of the grid-based models tested for calculating topographic depth. In the local slope allocation approach (left), each pixel in the lake is assigned the slope value of the closest pixel on shore and an explicit depth per pixel can be calculated by multiplying the tangent of its slope with the distance from shore. In the zonal slope allocation approach (right), all pixels in the lake are assigned the average slope value of all the pixels within a given buffer distance around the lake and an average depth is then calculated for the lake.

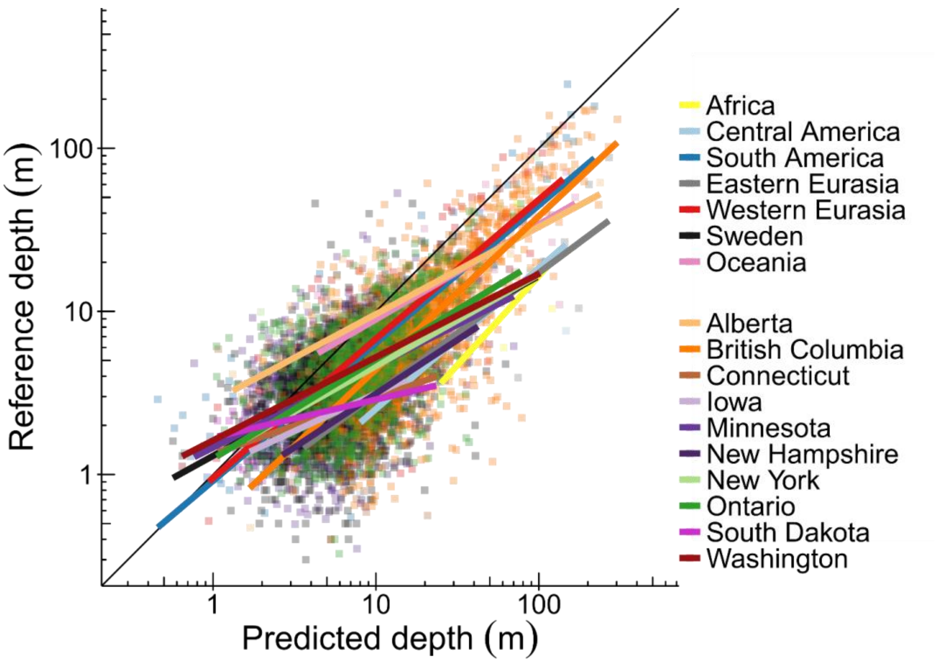

Supplementary Figure 3. Regional scatterplots of predicted topographic lake depth *vs.* reference mean depth.

The colored lines represent individual regressions for different regions derived by plotting modeled lake depth against the reference depths in the training dataset (7049 records). The black line is the identity line, a 1:1 relationship.

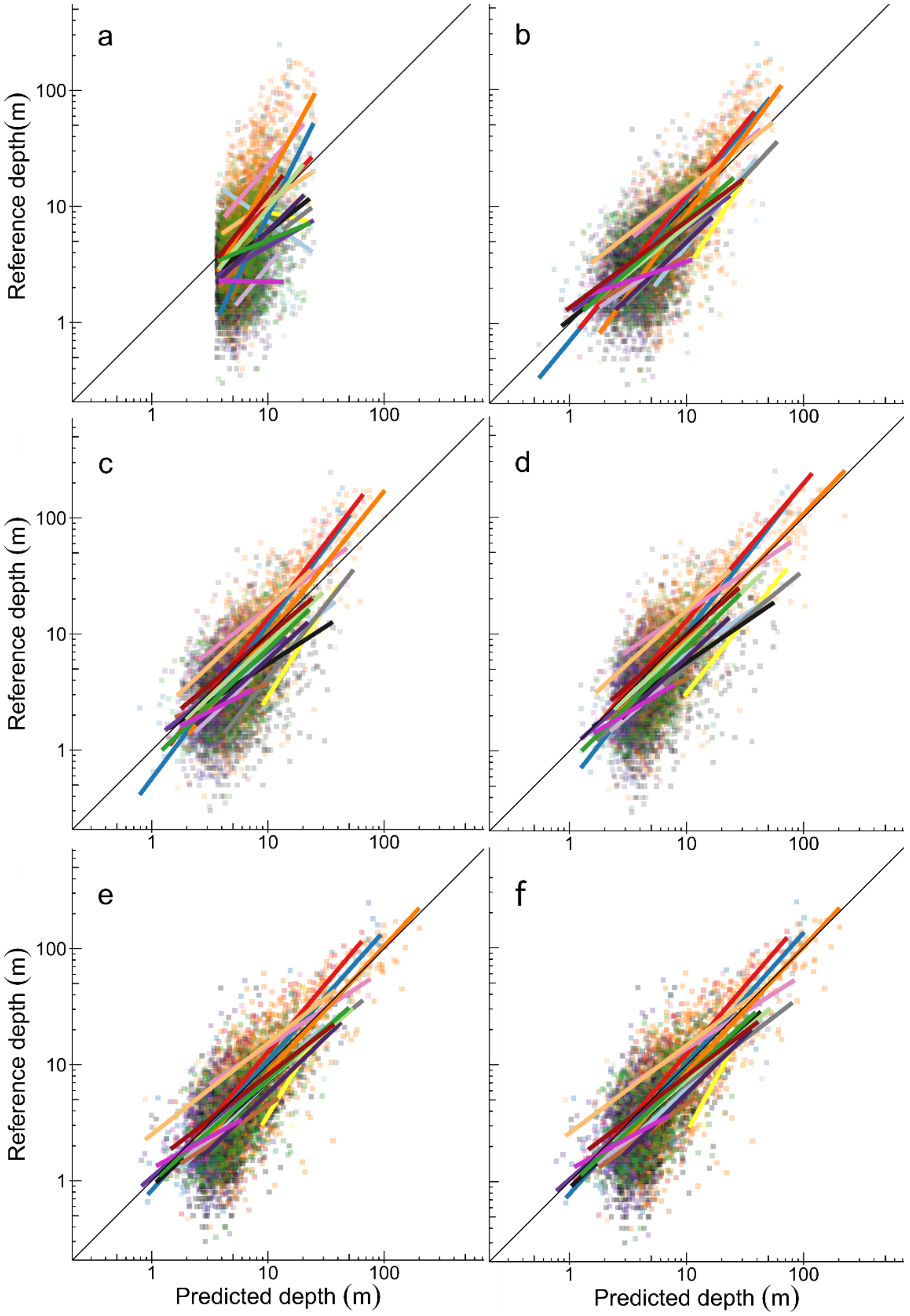

Supplementary Figure 4. Regional scatterplots of predicted *vs.* reference mean depth for the six best-fit statistical models tested in this study.

Panel (a) shows Model 1; (b) Model 2; (c) Model 3; (d) Model 4; (e) Model 5; (f) Model 6. See Supplementary Tables 4 and 5 for model equations and performance indices. The colored lines (for legend see Supplementary Fig. 3) represent individual regressions for different regions derived by plotting modeled lake depth against the reference depths in the training dataset (7049 records). The black line is the identity line, a 1:1 relationship.

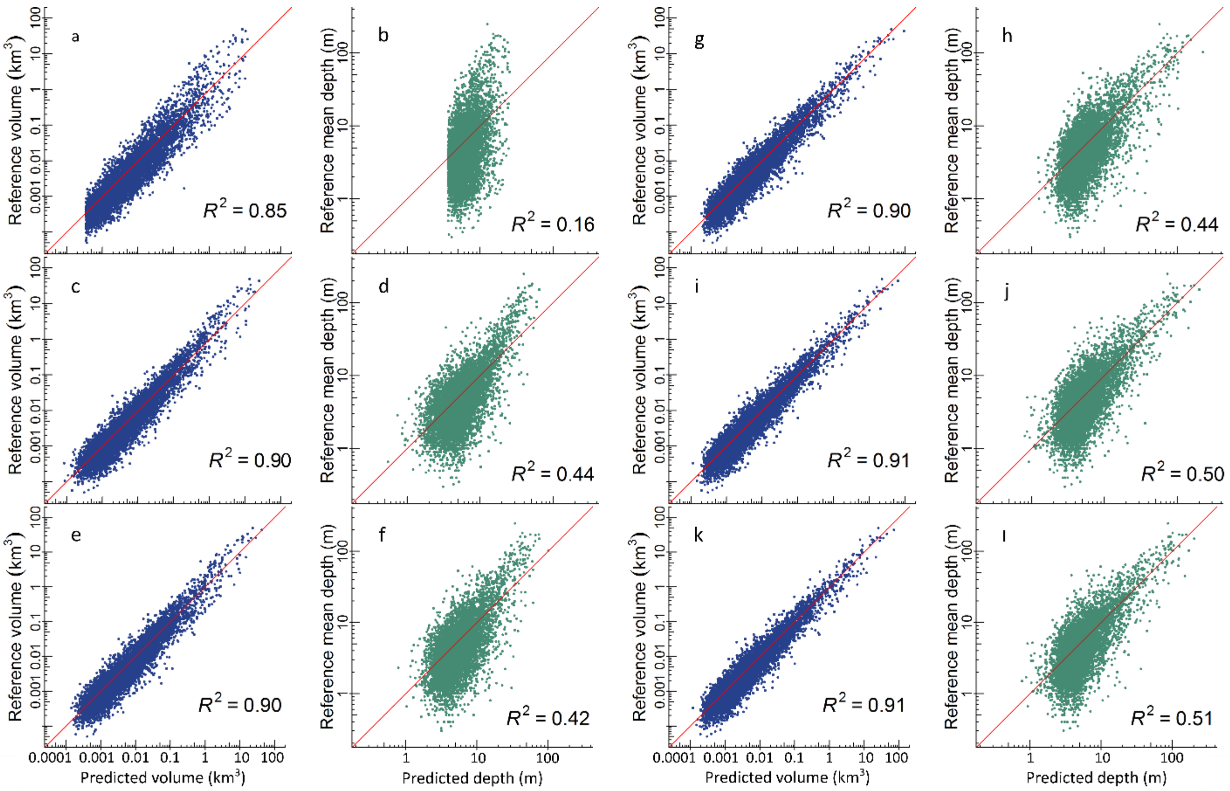

Supplementary Figure 5. Global scatterplots of predicted *vs.* reference lake volume and mean depth for the six best-fit statistical models tested in this study.

Panels (a&b) show Model 1; (c&d) Model 2; (e&f) Model 3; (g&h) Model 4, (i&j) Model 5; (k&l) Model 6. Individual panels show model results plotted against the reference values in the training dataset (7049 records). Blue dots (left panels) show predicted *vs.* reference lake volume; green dots (right panels) show predicted *vs.* reference mean depth. See Supplementary Tables 4 and 5 for model equations and performance indices. The straight cutoffs in Model 1 stem from the 10 ha threshold that applies to the only predictor variable of lake surface area. The red line is the identity line, a 1:1 relationship.

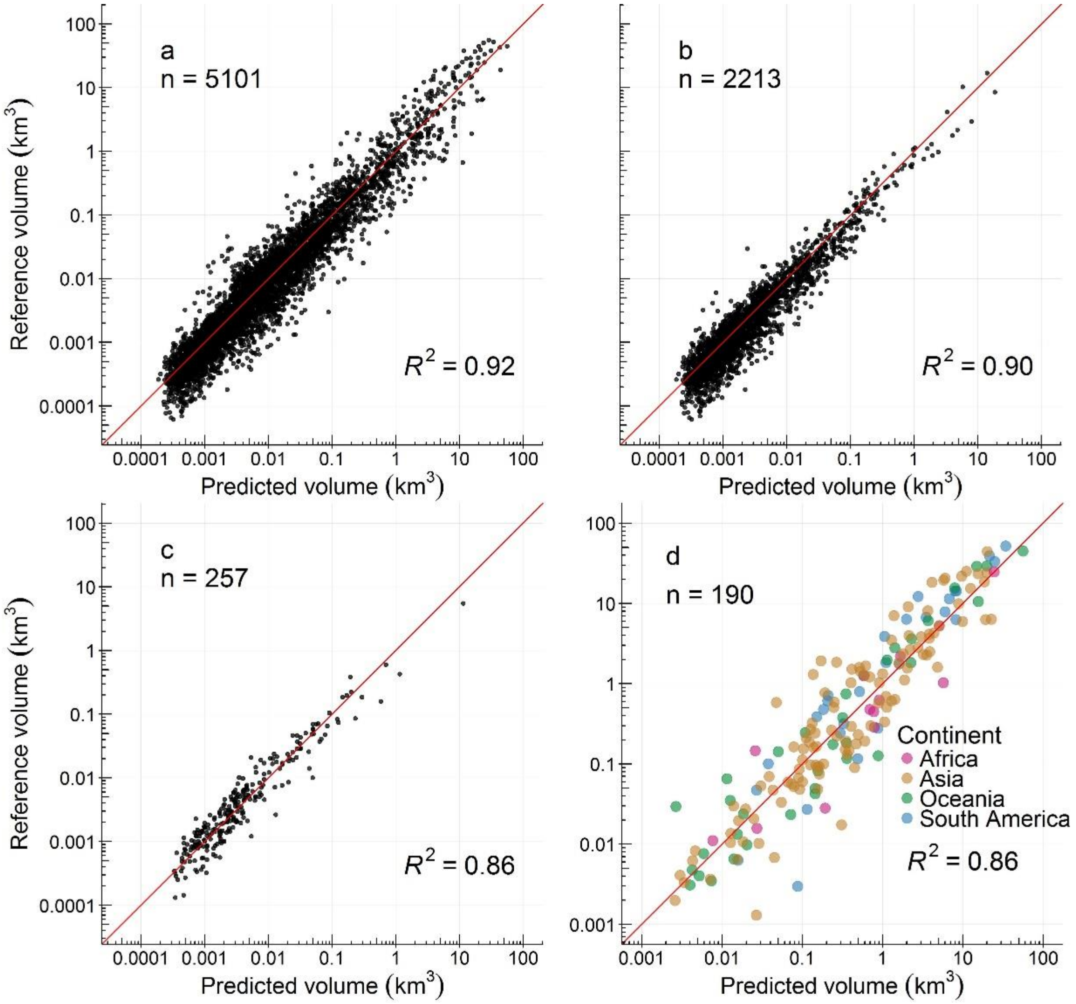

Supplementary Figure 6. Global and regional scatterplots of predicted *vs.* reference lake volume calculated by comparing Model 5 results with independent validation data.

Panel (a) shows data for all regions globally; (b) Sweden; (c) Quebec, Canada; (d) other continents. See Supplementary Table 2 for data sources. A slight overestimation is visible for small lakes in Sweden and Quebec, while a slight underestimation of the most voluminous (deep) lakes occurs outside of North America and Europe (panel d). Noticeably, as illustrated by the change in the axis scale of panel (d), lakes with available data outside of North America and Europe tend to be larger, as large lakes are more likely to be documented, and similar to deep lakes these larger lakes are difficult to model due to their increasingly unique characteristics. The red line is the identity line, a 1:1 relationship.

SUPPLEMENTARY TABLES

Supplementary Table 1. Overview of previous global estimates of total lake surface area and lake volume.

Year	Reference	Surface area (10^6 km^2)	Volume (10^3 km^3)
1894	Penck[1]	2.5 [a]	
1925	Thienemann (in Downing and Duart[2])	2.5 [a]	
1967	Nace[3]		229.0
1970	L'vovich[4]		230.0
1974	Tamrazyan[5]	2.7	166.0
1979	L'vovich[6]		275.0 [a]
1982	Mulholland and Elwood[7]	2.0 [a]	
1995	Meybeck[8]*	2.39 - 2.80 [a,b]	179.0 [a]
2003	Shiklomanov and Rodda[9]	2.06	176.4
2004	Lehner and Döll (GLWD)[10]*	3.20 [a]	
2006	Downing *et al.* [11]*	3.55 [a]	
2010	Ryanzhin *et al.* [12]	2.69 [a]	179.6 [a]
2011	Lewis Jr[13]*	3.10 [a]	
2012	McDonald *et al.* [14]*	3.53	
2013	Raymond *et al.* [15]**	2.74 [a]	
2014	Verpoorter *et al.* (GLOWABO)[16]***	4.76	
2016	**Messager *et al.* (HydroLAKES) – *this paper****	**3.23** [a]	**182.9** [a,c]

Values provided are for 'all lakes globally' unless size specifications are provided; volume estimates are not specified whether they include human-made reservoirs unless noted.

* including all lakes ≥0.01 km^2 (1 ha), based on statistical extrapolation for the smallest lakes

** including all lakes ≥0.001 km^2 (0.1 ha), based on statistical extrapolation for the smallest lakes

*** including all lakes ≥0.01 km^2 (1 ha), based on remote sensing imagery for entire dataset

[a] excluding human-made reservoirs

[b] depending on extrapolation method

[c] including all lakes ≥0.00001 km^3 (10,000 m^3), based on statistical extrapolation for the smallest lakes

Supplementary Table 2. Geographic location, quantity, type and source of reference lake depth data used for model training and validation.

	Region	Number of lakes	Data type	Source
Training	**Minnesota**	1362	Mean depth table	Division of Fish and Wildlife of the Minnesota Department of Natural Resources
	**British Columbia**	1351	Mean depth table	Ministry of Environment – Government of British Columbia
	**Ontario**	1005	Bathymetric lines	Ontario Ministry of Natural Resources
	**New Hampshire**	186	Bathymetric lines	New Hampshire Department of Environmental Services
	**Alberta**	166	Bathymetric raster	Alberta Geological Survey
	**Washington**	154	Bathymetric lines	Washington State Department of Ecology
	**New York**	127	Mean depth table	New York State Department of Environmental Conservation
	**Connecticut**	81	Bathymetric lines	Department of Energy and Environmental Protection
	**South Dakota**	74	Mean depth table	South Dakota Game Fish and Parks
	**Iowa**	23	Bathymetric lines	Iowa Geological Survey of the Iowa Department of Natural Resources
	**Sweden (training)**	2214	Mean depth table	Svenskt Vattenarkiv (SVAR) **-** Swedish Meteorological and Hydrological Institute
	**Europe other than Sweden**	165	Mean depth table	Waterbase - European Environment Agency
	**Other global**	141	Mean depth table	Peer-reviewed articles
	**Total (training)**	**7049**		
Validation	**Sweden (validation)**	2213	Mean depth table	Svenskt Vattenarkiv (SVAR) **-** Swedish Meteorological and Hydrological Institute
	**Quebec**	257	Mean depth table	Heathcote *et al.* [17]
	**Other global**	2631	Mean depth table	Global Lake Database (GLDB v2)[18,19]
	**Total (validation)**	**5101**		
	**TOTAL**	**12,150**		

Supplementary Table 3. Lake datasets used in the development of HydroLAKES.

Original dataset	Region	Original format and resolution	Number of lakes*
Canadian hydrographic dataset (CanVec)[20]	Canada (entire country)	Vector; 1:50,000	863,550
Shuttle Radar Topographic Mission (SRTM) Water Body Data (SWBD)[21]	56°S to 60°N	Raster; 1 arc-second (~30 m at the equator)	282,571
MODerate resolution Imaging Spectro-radiometer (MODIS) MOD44W water mask[22]	Russia above 60°N	Raster; 250 m	167,435
US National Hydrography Dataset (NHD)[23]	Alaska (entire state)	Vector; 1:24:000	58,496
European Catchments and Rivers Network System (ECRINS)[24]	Europe above 60°N and entire Norway	Vector; varying resolutions (~1:250,000)	50,699
Global Lakes and Wetlands Database (GLWD)[10]	World	Vector; 1:1 million	3,023
Global Reservoir and Dam database (GRanD)[25]	World	Vector; varying resolutions (1:1 million or better)	1,133
Other (own mapping)	World	Vector; varying resolutions (1:1 million or better)	781
**Total**			**1,427,688**

* The number of lakes refers to the polygons extracted from the source datasets; subsequent modifications to the polygon geometry may have been applied.

Supplementary Table 4. Equations of the six best-fit statistical models of increasing complexity tested in this study.

Model	Size class by area [km^2]	Best-fit equation	Residual variance s^2
**1**	**All**	$\log_{10}(D) = 0.6625 + 0.2289 \times \log_{10}(A)$	0.1150
**2**	**All**	$\log_{10}(D) = -0.0144 + 0.6887 \times \log_{10}(D_t)$	0.0765
**3**	**All**	$\log_{10}(D) = 0.0549 + 0.0774 \times \log_{10}(A) + 0.5893 \times \log_{10}(elv_{25\%})$	0.0790
**4**	**All**	n/a	n/a
	0.1-1	$\log_{10}(D) = 0.2045 + 0.0687 \times \log_{10}(A) + 0.4226 \times \log_{10}(elv_{25\%})$	0.0719
	1-10	$\log_{10}(D) = -0.0381 + 0.1315 \times \log_{10}(A) + 0.6488 \times \log_{10}(elv_{25\%})$	0.0814
	10-100	$\log_{10}(D) = -0.1535 - 0.0208 \times \log_{10}(A) + 0.8432 \times \log_{10}(elv_{25\%})$	0.0853
	100-500	$\log_{10}(D) = -0.3501 - 0.0024 \times \log_{10}(A) + 0.9216 \times \log_{10}(elv_{25\%})$	0.1044
**5**	**All**	n/a	n/a
	0.1-1	$\log_{10}(D) = 0.3826 + 0.1512 \times \log_{10}(A) + 0.4820 \times \log_{10}(S_{100})$	0.0678
	1-10	$\log_{10}(D) = 0.1801 + 0.2985 \times \log_{10}(A) + 0.8473 \times \log_{10}(S_{100})$	0.0689
	10-100	$\log_{10}(D) = 0.0379 + 0.2445 \times \log_{10}(A) + 1.1517 \times \log_{10}(S_{100})$	0.0692
	100-500	$\log_{10}(D) = 0.0123+ 0.2664 \times \log_{10}(A) + 1.1474 \times \log_{10}(S_{100})$	0.1094
**6**	**All**	n/a	n/a
	0.1-1	$\log_{10}(D) = 0.3346 + 0.1221 \times \log_{10}(A) + 0.3673 \times \log_{10}(S_{100}) + 0.1150 \times \log_{10}(D_t)$	0.0677
	1-10	$\log_{10}(D) = 0.0606 + 0.2158 \times \log_{10}(A) + 0.2808 \times \log_{10}(S_{100}) + 0.5771 \times \log_{10}(D_t)$	0.0678
	10-100	$\log_{10}(D) = -0.0692 + 0.0823 \times \log_{10}(A) + 0.7609 \times \log_{10}(S_{100}) + 0.4080 \times \log_{10}(D_t)$	0.0636
	100-500	$\log_{10}(D) = 0.0479 + 0.1260 \times \log_{10}(A) + 0.9462 \times \log_{10}(S_{100}) + 0.2350 \times \log_{10}(D_t)$	0.1071

D is the predicted mean depth in meters; A is the surface area of the lake in km^2; D_t is the topographic mean depth in meters; $elv_{25\%}$ is the difference in meters between lake surface and mean landscape elevation within a buffer width equal to 25% of the diameter of a circle that represents the lake area; and S_{100} is the average slope within a 100 m buffer around the lake in degrees. To avoid extreme outliers, minimum boundaries for mean depth were set (guided by histograms of depth frequency distributions and expert judgment): 0.5 m for lakes in the size classes 0.1-10 km^2, and 1 m for lakes in the size classes 10-500 km^2. Different color shades distinguish individual models and correspond with Supplementary Table 5.

Supplementary Table 5. Performance indices for mean depth and predicted volume for the six best-fit statistical models tested in this study.

Model	Size class by area [km^2]	R^2		RMSE		MAE		SMAPE [%]		Predicted Volume [km^3]		Reference volume [km^3]
		Mean	*95% CI*	Mean	*95% CI*	Mean	*95% CI*	Mean	*95% CI*	Mean	*95% CI*	
**1**	**All**	**0.16**		**10.96**		**4.60**		**64.1**		**520.5**		**913.8**
**2**	**All**	**0.44**		**8.51**		**3.53**		**50.4**		**590.6**		**913.8**
**3**	**All**	**0.42**		**8.25**		**3.61**		**48.9**		**699.8**		**913.8**
**4**	**All**	**0.44**		**7.28**		**3.45**		**48.3**		**980.6**		**913.8**
	0.1-1	0.25	*0.21-0.28*	3.70	*3.33-4.06*	2.36	*2.25-2.48*	48.0	*46.5-49.4*	5.9	*5.7-6.0*	5.9
	1-10	0.51	*0.47-0.54*	6.98	*6.15-7.81*	4.04	*3.78-4.30*	48.0	*46.4-49.7*	60.2	*57.7-62.8*	61.5
	10-100	0.72	*0.67-0.76*	19.62	*14.58-24.67*	10.06	*8.31-11.81*	49.3	*45.6-53.0*	282.1	*252.9-311.3*	293.4
	100-500	0.72	*0.61-0.83*	26.88	*9.21-44.54*	16.64	*8.36-24.92*	59.7	*48.0-71.4*	587.1	*424.0-750.2*	493.3
**5**	**All**	**0.50**		**6.42**		**3.12**		**47.4**		**913.1**		**913.8**
	0.1-1	0.24	*0.20-0.27*	3.71	*3.34-4.08*	2.38	*2.27-2.50*	48.3	*46.9-49.8*	5.8	*5.6-6.0*	5.9
	1-10	0.54	*0.51-0.57*	6.61	*6.00-7.22*	3.86	*3.62-4.10*	46.2	*44.7-47.8*	58.9	*56.5-61.2*	61.5
	10-100	0.75	*0.70-0.80*	17.73	*12.95-22.51*	8.64	*7.07-10.21*	47.2	*43.4-51.0*	286.3	*260.0-312.5*	293.4
	100-500	0.71	*0.59-0.83*	22.44	*10.22-34.66*	16.61	*9.73-23.50*	57.9	*45.6-70.2*	545.8	*397.2-694.5*	493.3
**6**	**All**	**0.51**		**6.31**		**3.07**		**47.2**		**942.7**		**913.8**
	0.1-1	0.24	*0.20-0.27*	3.70	*3.34-4.07*	2.38	*2.26-2.49*	48.3	*46.8-49.7*	5.8	*5.6-6.0*	5.9
	1-10	0.55	*0.51-0.58*	6.40	*5.81-6.94*	3.76	*3.52-3.99*	45.5	*43.9-47.1*	59.3	*57.0-61.6*	61.5
	10-100	0.77	*0.73-0.82*	17.29	*12.93-21.66*	8.53	*7.00-10.06*	42.7	*39.4-46.2*	280.3	*254.2-306.4*	293.4
	100-500	0.71	*0.59-0.83*	21.61	*9.70-33.53*	15.56	*8.49-22.64*	55.1	*41.7-68.4*	542.6	*402.1-683.2*	493.3

The 95% confidence intervals (CI) were obtained by bootstrapping with 10,000 replicates for each size class. Confidence intervals for predicted volume were obtained by applying the regression coefficients computed from each bootstrapping replication to the full training dataset (rather than to the sample used in that replication). When dimensional, performance indices are in meters. Different color shades distinguish individual models and correspond with Supplementary Table 4.

SUPPLEMENTARY DISCUSSION

Errors and uncertainties

Errors in the different datasets used in this study represent a substantial source of uncertainty. First, the bathymetric reference data can be erroneous due to insufficient sampling densities or imprecise measurement techniques. Additionally, errors in the reference data could stem from temporal changes in lake volumes and areas (both seasonally and due to long-term changes) or imprecision and inaccuracies in lake polygon generation. Second, the topographic data used in this study (EarthEnv-DEM90) include errors inherent in the underpinning digital elevation models; in particular, the Interferometric Synthetic Aperture Radar (InSAR) technology used for all areas below 60°N does not accurately reflect the "bald-Earth" surface but is affected by canopy height[26,27], while areas above 60°N are prone to other large errors[28].

The preferential availability of reference information for North America and Europe represents a bias towards these regions and towards mid- and high-latitude lakes in general. However, the USA, Canada, and Scandinavia comprise very variable landscapes, ranging from lowland plains to fjords and high mountains, and from subtropical to arctic climates. Also, Canada shows the highest concentration of lakes on Earth and is thus particularly representative. Nevertheless, we found slightly lower prediction errors for North America and Europe which may be attributed to this model bias. On the other hand, this observation may also be due to potentially lower quality of the reference data outside these regions.

Prediction errors remained generally low in our study, given the scale at hand, throughout all investigated regions globally. Compared to previous studies, including those by Pistocchi and Pennington[29] or Sobek *et al.* [30], we used a much larger global diversity and multitude of reference datasets in the model exploration, incorporating a broad range of lake types and regions, such that the bias in one dataset does not have an excessive influence on the overall predictions. However, using a broad width of datasets in a unified global approach is likely to introduce a tendency of harmonizing spatial differences and subduing regional heterogeneity. For example, mountain lakes, such as those found in the European Alps, British Columbia, and the Andes in South America, tend to be underestimated (see Supplementary Fig. 1) while shallow lakes of low-land areas tend to be overestimated.

SUPPLEMENTARY REFERENCES

1 Penck, A. *Morphologie der Erdoberfläche* Vol. 2 (J. Engelhorn, 1894).
2 Downing, J. A. & Duarte, C. M. in *Lake Ecosystem Ecology: A Global Perspective* (ed. Likens, G. E.) 220–229 (Academic Press, 2009).
3 Nace, R. L. Are we running out of water? Report No. 536, (U.S. Geological Survey, 1967).
4 L'vovich, M. I. World Water Balance (General Report). 401-415 (IASH-UNESCO, 1970).
5 Tamrazyan, G. Total lake water resources of the planet. *Bull. Geol. Soc. Finland* **46**, 23-27 (1974).
6 L'vovich, M. I. *World Water Resouces and Their Future*. (American Geophysical Union, 1979).
7 Mulholland, P. J. & Elwood, J. W. The role of lake and reservoir sediments as sinks in the perturbed global carbon cycle. *Tellus* **34**, 490-499, doi:10.1111/j.2153-3490.1982.tb01837.x (1982).
8 Meybeck, M. in *Physics and chemistry of lakes* (eds Lerman, A., Imboden, D. M. & Gat, J. R.) 1-35 (Springer, 1995).
9 Shiklomanov, I. A. & Rodda, J. C. *World water resources at the beginning of the twenty-first century* Vol. 13 (Cambridge University Press, 2003).
10 Lehner, B. & Döll, P. Development and validation of a global database of lakes, reservoirs and wetlands. *J. Hydrol.* **296**, 1-22, doi:10.1016/j.jhydrol.2004.03.028 (2004).
11 Downing, J. A. *et al.* The global abundance and size distribution of lakes, ponds, and impoundments. *Limnol. Oceanogr.* **51**, 2388-2397, doi:10.4319/lo.2006.51.5.2388 (2006).
12 Ryanzhin, S. V., Subetto, D. A., Kochkov, N. V., Akhmetova, N. S. & Veinmeister, N. V. Polar lakes of the World: Current data and status of investigations. *Water Resour.* **37**, 427-436, doi:10.1134/S0097807810040019 (2010).
13 Lewis Jr, W. M. Global primary production of lakes: 19th Baldi Memorial Lecture. *Inland Waters* **1**, 1-28, doi:10.5268/IW-1.1.384 (2011).
14 McDonald, C. P., Rover, J. A., Stets, E. G. & Striegl, R. G. The regional abundance and size distribution of lakes and reservoirs in the United States and implications for estimates of global lake extent. *Limnol. Oceanogr.* **57**, 597-606, doi:10.4319/lo.2012.57.2.0597 (2012).
15 Raymond, P. A. *et al.* Global carbon dioxide emissions from inland waters. *Nature* **503**, 355-359, doi:10.1038/nature12760 (2013).
16 Verpoorter, C., Kutser, T., Seekell, D. A. & Tranvik, L. J. A global inventory of lakes based on high-resolution satellite imagery. *Geophys. Res. Lett.* **41**, 6396-6402, doi:10.1002/2014GL060641 (2014).
17 Heathcote, A. J., del Giorgio, P. A., Prairie, Y. T. & Brickman, D. Predicting bathymetric features of lakes from the topography of their surrounding landscape. *Can. J. Fish. Aquat. Sci.* **72**, 1-8, doi:10.1139/cjfas-2014-0392 (2015).
18 Choulga, M., Kourzeneva, E., Zakharova, E. & Doganovsky, A. Estimation of the mean depth of boreal lakes for use in numerical weather prediction and climate modelling. *Tellus A* **66**, doi:10.3402/Tellusa.V66.21295 (2014).

19 Kourzeneva, E., Asensio, H., Martin, E. & Faroux, S. Global gridded dataset of lake coverage and lake depth for use in numerical weather prediction and climate modelling. *Tellus A* **64**, doi:10.3402/tellusa.v64i0.15640 (2012).
20 Natural Resources Canada. CanVec Hydrography: Waterbody Features. Version 12.0. Data available online (ftp://ftp2.cits.rncan.gc.ca/pub/canvec/), accessed May 2013 (2013).
21 Slater, J. A. *et al.* The SRTM data “finishing” process and products. *Photogramm. Eng. Rem. S.* **72**, 237-247, doi:http://dx.doi.org/10.14358/PERS.72.3.237 (2006).
22 Carroll, M. L., Townshend, J. R., DiMiceli, C. M., Noojipady, P. & Sohlberg, R. A. A new global raster water mask at 250 m resolution. *Int. J. Digital Earth* **2**, 291-308, doi:10.1080/17538940902951401 (2009).
23 USGS. National Hydrography Geodatabase: Alaska, data available via http://nhd.usgs.gov/ or The National Map viewer (http://viewer.nationalmap.gov/viewer/nhd.html?p=nhd), accessed July 2013 (2013).
24 European Environment Agency. ECRINS (European Catchments and RIvers Network System): Lakes. Version 1 (2012).
25 Lehner, B. *et al.* High-resolution mapping of the world's reservoirs and dams for sustainable river-flow management. *Front. Ecol. Environ.* **9**, 494-502, doi:10.1890/100125 (2011).
26 Brown, C. G. *Tree height estimation using Shuttle Radar Topography Mission and ancillary data.* PhD dissertation, University of Michigan, Department of Electrical Engineering and Computer Science (2003).
27 Kellndorfer, J. *et al.* Vegetation height estimation from shuttle radar topography mission and national elevation datasets. *Remote Sens. Environ.* **93**, 339-358, doi:10.1016/j.rse.2004.07.017 (2004).
28 Tachikawa, T. *et al.* ASTER Global Digital Elevation Model Version 2–Summary of Validation Results (Ministry of Economy, Trade, and Industry (METI) of Japan and the United States National Aeronautics and Space Administration (NASA), 2011).
29 Pistocchi, A. & Pennington, D. European hydraulic geometries for continental SCALE environmental modelling. *J. Hydrol.* **329**, 553-567, doi:10.1016/j.jhydrol.2006.03.009 (2006).
30 Sobek, S., Nisell, J. & Fölster, J. Predicting the volume and depth of lakes from map-derived parameters. *Inland Waters* **1**, 177-184, doi:10.5268/IW-1.3.426 (2011).
